# Near misdiagnosis of acute HIV-infection with ELISA-Western Blot scheme: Time for mindset change

**DOI:** 10.1016/j.idcr.2021.e01168

**Published:** 2021-05-21

**Authors:** Carlos Eduardo Medina-De la Garza, María de los Ángeles Castro-Corona, Mario César Salinas-Carmona

**Affiliations:** a Immunology Service, School of Medicine and University Hospital “Dr. José E González”, Universidad Autónoma de Nuevo León, Av Gonzalitos 235, Mitras Centro, Monterrey 64460, Mexico; b Center for Research and Development in Health Sciences, CIDICS, Universidad Autónoma de Nuevo León. Av Gonzalitos s/n Mitras Centro, Monterrey 64460, Mexico

**Keywords:** HIV-misdiagnosis, ELISA-Western blot, HIV-discordant results, Acute HIV infection, HIV-diagnosis counseling, Point of care HIV-testing

## Abstract

Some HIV-infection diagnostic guidelines and health care providers still rely on the ELISA-Western blot diagnostic algorithm. We present a near misdiagnosis case with discordant test results and a lack of proper counseling. We point out the need for an assertive update of health care providers on diagnostic HIV-tests

## Highlights

• Many health care providers still rely upon the ELISA-Western blot scheme for HIV-diagnosis.
• Western blot may fail to detect an acute HIV-infection.
• Point of Care settings using rapid tests should consider anamnesis and patient risk assessment for an accurate HIV-Testing.
• Discordant HIV-testing results require knowledgeable counseling.
• Health care providers should be aware and updated about changes in HIV-testing guidelines.

## Introduction

Current guidelines for diagnosing HIV infection in Mexico require a confirmatory Western blot (WB) after a positive ELISA test [1]. Serological detection of anti-HIV antibodies during acute infection takes three to six weeks after infection due to the window period (WP) [2]. The diagnostic algorithm introduced by CDC in 2014 recommends the fourth-generation HIV-EIA test (4th Gen), which detects both antibodies and p24 antigen [3,4] and reduces WP to two weeks after infection [5]. However, health care providers who still follow guidelines based on ELISA/WB scheme may not consider a reactive 4th Gen EIA/ELISA to be true positive unless confirmatory WB is also positive [1,5]. This situation may lead to patients' misdiagnosis in the early seroconversion period, where antibodies are not detected by WB [2]. The World Health Organization (WHO) recommended abandoning WB use in favor of EIA and rapid tests (RT) [6,7] to achieve the goals of an accurate and reliable diagnosis globally, providing comprehensive HIV-testing services to increase the number of people aware of their infection status [7]. It is estimated that in 2018 nearly 85 % of people in eastern and southern Africa and 80 % of people with HIV worldwide knew their status [7]. Despite great advances in diagnosis by the use of RT and availability of therapy, in many low and middle-income countries (LMIC), significant misdiagnosis causes remain, such as performance of suboptimal testing algorithms, poor diagnosis delivery, inadequate initial assessment, and error interpreting results [8,9]. Additionally, it is suggested that in LMIC, appropriate testing strategies should be established for the diagnosis of acute HIV infection (AHI), for which explicit diagnostic algorithms, point of care testing, and clinical risk-scores may be critical [10]. Here, we present an illustrative case of a near-misdiagnosis in a patient in early seroconversion who endured several rapid and laboratory-based ELISA HIV-testing with discordant results and referred to our laboratory for WB-testing.

## Case report

A 29-year-old male presented to a general practitioner with pain in the left arm and legs, weakness, sore throat, and chills. He was diagnosed with pharyngitis and prescribed antibiotics and paracetamol. The next day he developed 38.5 °C fever with intense diaphoresis and consulted with another general practitioner. The physician confirmed pharyngitis, endorsed symptomatic treatment but withdrew antibiotics. The patient remained febrile and weak for the next five days. Unsolicited and unsupervised laboratory tests were performed, including a rapid HIV-antibody test which was reported as non-reactive. He had had a rapid HIV test two months before the onset of symptoms, which was negative. The WBC count and general labs showed leukopenia (3.9 × 10^3^/μL) with 42 % neutrophils (1.65 × 10^3^/μL), 42 % lymphocytes (1.68 × 10^3^/μL), 14 % Monocytes (0.58 × 10^3^/μL) and platelets 107 × 10^3^/μL. Total cholesterol 105 mg/dL, alanine aminotransferase 134 UI/L, aspartate aminotransferase 190 UI/L, and lactate dehydrogenase 307 UI/L.

Seven days after the beginning of symptoms, fever subdued, but malaise remained. Fearing an HIV-infection because of a previous history of risk behavior, the patient sought laboratory-based testing. A 4th Gen chemiluminescence HIV-test was performed and reported reactive. After this result, delivered without counseling, he requested further HIV testing in three different places the same day: 1) Public Health Care Provider in a primary care setting, where a 3rd Gen-rapid test reported non-reactive, 2) Public AIDS-Counseling Center where a 3rd Gen- rapid test reported non-reactive, and 3) an AIDS-NGO facility where two rapid tests (Genie-Fast HIV1/2 3rd- and ALERE HIV-Combo, 4th-Gen) were performed simultaneously, and both reported non-reactive. The next day, he asked for primary care consultation and underwent another rapid 3rd Gen-test, reported non-reactive.

In doubt, the patient returned to the laboratory where he first tested positive, and a new test reported reactive again. The primary care physician ordered a WB in a reference laboratory. The WB was negative, but the clinical and testing history rose suspicion. We suggested and performed a physical examination of the patient and found prominent cervical bilateral adenopathy. Following detailed anamnesis, the patient reported unprotected receptive sex with male partners A and B, respectively, eight and six weeks before the symptoms. As he first tested positive, he requested both partners to undergo the same laboratory-based testing, and both were reported negative. The only suspected infection source was non-consensual, unprotected sexual contact ten days before the beginning of symptoms. The referring primary care physician considered the WB-result a true negative. However, because of the two reactive lab-based tests, the clinical picture and anamnesis, we suggested a further 4th Gen test (Genscreen Ultra HIV, Biorad) in our reference laboratory, which was reactive. Thus, eleven days after the onset of symptoms, five rapid non-reactive tests, one negative WB, and three 4th Gen reactive tests, the patient was diagnosed with acute retroviral syndrome and ongoing seroconversion. He was referred to a Public Health Institution, where another lab-based 4th Gen test was reactive, and a quantitative HIV-1 RNA viral load test reported > 3,000,000 copies/mL.

## Discussion

Roughly 29–69 % of persons with AHI may seek healthcare attention for symptoms following HIV infection [10]. This fact represents an opportunity for detection if an oriented clinical history is made and enough diagnostic suspicion exercised. In our case, the patient was examined by two different primary care physicians who may have observed only nonspecific symptoms and diagnosed and treated uncomplicated pharyngitis without recommending any further general or specific lab testing. There was no skin rash, and the other symptoms and signs observed did not raise suspicion of HIV infection; therefore, no risk assessment was performed, and the infection remained unrecognized. Indeed, AHI diagnosis is difficult because of the unspecific clinical picture, its brief duration, and the initial absence of detectable antibodies [5,10]. The relevance of accurate diagnosis during AHI and the early infection stage (up to six months after infection) lies in the significant clinical and immuno-virological benefit for the patient when linked to early antiretroviral therapy and of epidemiological significance by helping reduce transmission risk [5,10].

On the other hand, in Mexico, for an HIV-laboratory-based test, written order by a physician and informed consent is mandatory, but in point-of-care settings, it is not. In any setting, however, specific counseling before or after HIV testing is not obligatory. In our case, the 4th Gen laboratory-based test performed seven days after onset of symptoms provided an accurate reactive result. Nevertheless, this correct lab diagnosis was shadowed because the test result was not delivered with proper counseling or linkage to care. Afterward, the four rapid tests performed were non-reactive, which increased patients' perception of a possible diagnostic failure. Point of care health personnel either ignored or disregarded the reactive lab-based test and overlooked suggestive symptoms and possible recent exposure.

Close supervision and precise diagnostic advice are required to prevent misdiagnosis and give a reliable interpretation of discordant results, particularly in recent exposure and suspicion of AHI. Kufa et al. in South Africa identified acute or early HIV infection as a factor for false-negative results [11]. Health care providers in primary or point-of-care settings should be updated regarding the optimal performance of laboratory-based 4th Gen tests and know the limitations and timing of rapid tests regarding WP and AHI [12,13]. HIV-Testing services, including rapid testing in resource-limited settings and LMIC, have an additional challenge posed by a lack of guidelines, training, and quality assurance monitoring, among others [14]. These shortcomings have been reviewed by Johnson et al. [8,9]. They examined the origins and cause of HIV misdiagnosis and testing errors in LMIC at the patient, provider, facilities, and system level. They support the use of algorithms and strategies validated at the country or regional level, performed by trained and supervised staff [8,9].

The 2014 CDC recommendations were advanced to avoid negative or indeterminate WB in acute infections, reduce WP, provide early diagnosis and allow prompt treatment [3,4]. WHO consolidated guidelines further point out the clinical, economic, and social disadvantages of WB use [6,7]. In our case, WB use as a tie-breaker led to a near misdiagnosis and potential delay for linkage of care. In our country, current guidelines still require a WB confirmation despite a reactive 4th Gen test [1]. Although many health care providers use CDC recommendations, the longstanding use of the ELISA-WB diagnostic scheme may still be present in the diagnostic approach of many of them [5] and used in different geographical settings around the globe [6].

The diagnosis of acute HIV infection requires high clinical suspicion and judgment. Thus, anamnesis on recent risk behavior and symptoms are essential, as is the proper use of the best diagnostic tests available. Local regulatory boards should endorse updated guidelines to avoid misdiagnosis of acute infection and provide uniform procedures to encourage compliance with CDC, WHO, and other agencies, and abandon Western blot's diagnostic role in HIV [3,6,15]. Primary care health personnel should be assertively advised of the change in the procedures and endorsement of a new HIV-diagnostic mindset.

## Conflicts of interest

None.

## Funding

This research did not receive any specific grant from funding agencies in the public, commercial, or not-for-profit sectors.

## Consent

No personal information or identifying details of patient are used. There is Informed consent on the requested performance of HIV diagnostic tests in our lab

## Ethical approval

N/A.

## Author contribution

All authors approved the final version of the manuscript

## CRediT authorship contribution statement

**Carlos Eduardo Medina-De la Garza:** Conceptualization, Formal analysis, Writing - original draft, Writing - review & editing. **María de los Ángeles Castro-Corona:** Conceptualization, Formal analysis, Writing - review & editing. **Mario César Salinas-Carmona:** Conceptualization, Formal analysis, Writing - review & editing.
